# Leucorrhœa

**Published:** 1892-04

**Authors:** 


LEUCORRHŒA.—Prof. C. Braun, of Vienna, recommends in leucorrhœa, associated with chlorosis, the following:

R. Ferri sulph. cryst.,
Kali carbonic. et. tartara, aa dr. j 1-4.
Extr. et pulv. r. gentianæ q. s. et fiant pil. No. 60.

Sig.—Two pills, morning, noon and evening.

If the lucorrhœa be bloody, then:

R. Ferri sulph. cryst.,
Kali hydrocarbon, aa dr. j.
Ergotin pur., gr. xxiij.
Extr. et pulv. r. liqueritiæ, aa q. s. et f. pil. No. 50.

Sig.—Two or three pills, morning, noon and evening.

If constipation be present, then:

R. Ferri sulph. cryst.,
Kali hydrocarbon, aa dr. j.
Aloes lucid., gr. xxx.

Sig.—A teaspoonful, morning, noon and evening.

Or:

R. Ferri oxidat. dialyzat., dr. j. 1-4.
Aq. f. d., f. dr. iv.
Syrup. rub. idæi, f. dr. vj.

Sig.—A teaspoonful, morning, noon and evening.

These last two formulæ are well borne by the stomach.—*Clinic of Prof. C. Braun, Vienna.*—(*Annals of Gynæcology.*)

